# AdaBoost Ensemble Methods Using K-Fold Cross Validation for Survivability with the Early Detection of Heart Disease

**DOI:** 10.1155/2022/9005278

**Published:** 2022-04-18

**Authors:** T. R. Mahesh, V. Dhilip Kumar, V. Vinoth Kumar, Junaid Asghar, Oana Geman, G. Arulkumaran, N. Arun

**Affiliations:** ^1^Department of Computer Science and Engineering, Faculty of Engineering and Technology, JAIN (Deemed-to-be University), Bangalore, India; ^2^Department of Computer Science and Engineering, Vel Tech Rangarajan Dr. Sagunthala R&D Institute of Science and Technology, Chennai, India; ^3^Faculty of Pharmacy, Gomal University, Dera Ismail Khan 29050, Khyber Pakhtunkhwa, Pakistan; ^4^Stefan Cel Mare University of Suceava, Suceava, Romania; ^5^Department of Electrical and Computer Engineering, Bule Hora University, Bule Hora, Ethiopia

## Abstract

As a result of technology improvements, various features have been collected for heart disease diagnosis. Large data sets have several drawbacks, including limited storage capacity and long access and processing times. For medical therapy, early diagnosis of heart problems is crucial. Disease of heart is a devastating human disease that is quickly increasing in developed and also developing countries, resulting in death. In this type of disease, the heart normally fails to provide enough blood to different body parts in order to allow them to perform their regular functions. Early, as well as, proper diagnosis of this condition is very critical for averting further damage and also to save patients' lives. In this work, machine learning (ML) is utilized to find out whether a person has cardiac disease or not. Both the types of ensemble classifiers, namely, homogeneous as well as heterogeneous classifiers (formed by combining two separate classifiers), have been implemented in this work. The data mining preprocessing using Synthetic Minority Oversampling Technique (SMOTE) has been employed to cope with the imbalance problem of the class as well as noise. The proposed work has two steps. SMOTE is used in the initial phase to reduce the impact of data imbalance and the second phase is classifying data using Naive Bayes (NB), decision tree (DT) algorithms, and their ensembles. The experimental results demonstrate that the AdaBoost-Random Forest classifier provides 95.47% accuracy in the early detection of heart disease.

## 1. Introduction

Heart disease is mainly observed as the world's most dangerous and life-threatening chronic disease. During heart illness, the heart generally fails to deliver enough blood to different body regions so as to allow them to operate normally. The narrowing and occlusion of coronary arteries can cause heart failure. Heart disease is one among the leading reasons for death nowadays across the globe [1]. This leads to crucial requirement of monitoring the functioning organs in the human body and a critical aspect in monitoring health records of cardiovascular system. The coronary arteries control the entire circulation of blood to the heart. According to the latest survey, United States is one of the severely affected countries with relatively high ratio of heart disease observed in patients. The symptoms like breathing problem, physical body weakness, exhaustion, and swollen feet among various other symptoms are the most typical markers of heart disease [2]. Most of the cardiovascular diseases affecting people across the world are usually fatal. So, to overcome this problem, development of new technique may aid in detection of heart diseases in early stages as there is huge growth in the technology. Also, before causing substantial damage to avoid advantageous problems in terms of time, cost, and saving human lives machine learning techniques are used to focus on monitoring the heart diseases. Machine learning involves emerging techniques in manipulating and extracting features or relevant data information in possible way [3]. Machine learning is one of the complex fields and also has huge scope in various applications which is expanding all the time. Machine learning techniques consist of supervised learning, unsupervised learning, and also ensemble learning classifiers, which are mainly used to forecast the heart diseases in early stages with increase in accuracy results [4].

In the past years, academicians and researchers attempted to create and implement many intelligent programs by applying predefined procedures, which are similar to regular existing program works [2]. But, still there is a lag in monitoring many observations and instances in timely manner to overcome many societal challenges. Nowadays, very challenging tasks include photo tagging, identification of web-based ranking, identification of spam, or no spam Emails. To overcome these tasks or objectives, one of the options includes development of a program generating relevant rules to evaluate the data samples. It is also called training set, and one of the common emerging fields used for this is machine learning methods. Since 2010–2015, many intelligence software-based machine learning methods are applied including recognition systems on patient images to improve the accuracy results from 72% to 95% [5].

Most of the machine learning applications are evolving in present days and affecting every aspect in our daily lives. Machine learning is applicable in many emerging areas like healthcare monitoring systems, pattern recognition and feature extraction, text and speech recognition, education systems, military and defense applications, fraud detection, etc. Artificial intelligence takes the main lead in the development of ML technology systems. ML technology also simulates human learning systems from the input dataset or information. Many machine learning algorithms from firms such as Facebook, Amazon, or Flipkart are boosting the business trends in developing various brands [6]. With the help of past data or information, machine learning tries to discover new patterns in applying algorithms to achieve feasible outcome results. Also adding value to the business trends or organizations mainly focuses on monitoring future situations and outcome [7].

## 2. Related Work

A lot of research work is carried out using machine learning methods in achieving more accurate results and predicting outputs based on input dataset [8]. Machine learning plays a very important role in view of new trends and new techniques based on customer behavior or various input patterns, in the development of new products and new brands [9]. Enterprises can understand the customer needs at deeper level to overcome their needs using machine learning algorithms depending, for various applications, on their outcomes [10]. Machine learning also increases the importance in business operations and artificial intelligence is becoming practically high using today's ML models.

One of the new strategies for detecting cardiac diseases, mainly based on Co-Active Neuro-Fuzzy Interference Systems (CANFIS), is applied in one of the research work [11]. Most of the research study is based on regularity in detection of heart diseases based on their strategies as well as on their difficulties. Classifier strategies for the detection of heart diseases are demonstrated using machine learning algorithm, Naive Bayes classifier model. Most of the survey is carried out on various applications, in many research papers, by using data mining algorithms for prediction of heart diseases [12]. But traditional invasive-based approach is carried out using machine learning algorithms. The classifier models for diagnosing heart diseases are based on medical history of patients, patient test results, or scan results so that researchers or doctors can research on connected symptoms [13].

Alternatively, one more disadvantage is that the dye used is harmful as it affects kidneys, as it increases creatinine, including a high cost, a different kind of adverse effects, and a very good level of technological knowledge [14]. The traditional method is comparatively costly and also computationally intensive method for disease diagnosis which takes time to assess [15]. Researchers have tried to create various noninvasive smart healthcare systems which are based on predictive ML techniques, namely, SVM, K-NN, Naive Bayes (NB), and, also, decision tree (DT), among others, to overcome the challenges in conventional invasive-based methods for the identification of heart disease [16].

In the medical field, one of the most used classifiers is the decision tree. In this work [17] SEER medical datasets were used to predict the disease survivorship using classification and regression trees (CART).

In this work [18], use of neural networks was introduced to diagnose and forecast heart disease as well as blood pressure. A Deep Neural Network was built using the given disease attributes to generate an output that was accomplished by the output perceptron and almost included 120 hidden layers, which is the most basic and relevant method for ensuring an accurate result of having heart disease if the model is using the test dataset [19]. The use of a supervised algorithm for cardiac disease diagnostics is being recommended [17]. When the attributes of data are associated, the random forest approach has a tendency to favor the smaller group [20]. This is why, in order to alleviate the challenge of imbalanced data and limit the probability of bias against minorities in the dataset, the SMOTE method is being used. In this study [21], a combination of SMOTE and Artificial Neural Network (ANN) has been used to diagnose ovarian cancer using a publicly available dataset of ovarian cancer. The research demonstrates that, by using the preprocessing methodology of SMOTE to decrease the impact of data imbalance, we can improve the performance and efficiency of neural networks in cancer classification. On large datasets, most single classifier algorithms have the drawbacks of being computationally expensive and difficult. For large datasets, in particular, classification approaches do not give consistent and reliable results, making some individual classifier systems wasteful and unreliable [22]. For example, the DT approach is particularly good at managing intervariable interactions, but it struggles with linear relationships between variables [23].

In recent years, ensemble classifiers have become a popular strategy in machine learning and pattern recognition. In a nutshell, it is a method for combining the findings of many classifiers. The ensemble method's main goal is to improve classification efficiency by weighing multiple independent classifiers and thereby combining them into a single or an individual classifier that outperforms each one individually [22, 24, 25].

## 3. Exploratory Knowledge

One of the most well-known areas of medical research is the research for heart disease. Early identification and accurate projections of heart diseases have a significant impact on therapy and reduce patient mortality rates. The sections that follow provide brief descriptions of the algorithms used to detect heart disease in this study.

### 3.1. Decision Tree (DT) Classifier

A decision tree is a supervised ML algorithm that makes decisions based on a set of rules, very similar to how normally people do. A ML classification method is designed to make judgments, in one sense. Classification and regression problems can both be solved with this classifier [26].

There are different notions that define the model. They are given below.

(i) Entropy: Entropy is a measurement of a system's unpredictability or disorder. In the year 1850, a German physicist named Rudolf Clausius proposed this hypothesis. It is computed as shown in
  (1) $$\begin{array}{c} \text{Entropy}=-\sum p\left(X\right)\log\;p\left(X\right), \end{array}$$
where p(X) is a fraction of examples in a given class.
(ii) Gini Index: It is also called the Gini coefficient, which is a measure of income distribution in a population. Corrado Gini, an Italian statistician, created it in 1912. The Gini impurity is computed using
  (2) $$\begin{array}{c} \text{Gini Inpurity}=1-\sum_{i=1}^{C}{\left(p_{i}\right)}^{2}. \end{array}$$
(iii) Information Gain: The reduction in entropy achieved by changing a dataset is known as information gain, and it is frequently utilized in the training of decision trees. The entropy of a dataset before and after a transformation is used to calculate information gain. It is computed using
  (3) $$\begin{array}{c} IG\left(D_{p},f\right)=I\left(D_{p}\right)-\frac{N_{\text{left}}}{N}I\left(D_{\text{lef}t}\right)\_\frac{N_{\text{right}}}{N}I\left(D_{\text{right}}\right), \end{array}$$
where *f* is feature split on *D*_*p*_which is parent dataset; *D*_left_ is left child node dataset;*D*_right_ is right child node dataset; I is impurity criterion; N is total number of samples; *N*_left_ is samples number of left child node; *N*_right_ is samples number of right child node.

### 3.2. The CART Algorithm

The CART algorithm was first introduced by Breiman et al. [27]. Hunt's algorithm is used to create the CART. To build a DT, it can process categorical as well as continuous attributes. It also accounts for missing data and constructs the DT by making use of Gini Index as an attribute selection criterion. CART divides the given datasets (training set) into binary segments and builds binary trees as a result. The Gini Index is not employed in the ID3 and C4.5 probabilistic assumptions. In order to increase accuracy of classification, CART algorithm increases the accuracy by making use of cost-complexity pruning for removing unpredictable branches from the DT.

### 3.3. Alternating Decision Tree (AltDTree)

AltDTree is a classification ML method. It is related to boosting and generalizes decision trees. An AltDTree is made up of a series of decision nodes that indicates a predicate condition and prediction nodes that hold a single number [28]. Classic DTs, Voted DTs, and Voted Decision Stumps are all generalized into AltDTree. It allows any boosting implementation to extract the AltDTree model from the data as a learning method. In the context of the decision tree, AltDTree is an appealing extension of boosting. It enables the use of various boosting strategies to create an AltDTree model with unique properties that can handle a wide range of applications.

### 3.4. Random Forest (RF) Classifier

RF works by using the training data to create several decision trees. In the case of classification, every tree suggests output as a class; also the class with greatest number of outputs is selected as the final outcome [29]. In order to build, number of trees must be specified. RF is such a technique for aggregating or even bagging bootstrap data. This method is used to reduce an important parameter called variance in the outcomes.

### 3.5. Reduced Error Pruning Tree (RedEPTree)

Top-down induction of decision trees has been observed to be hampered by the pruning phase's poor performance. It is known, for example, that the size of the resulting tree rises linearly with the sample size, despite the fact that the tree's accuracy does not improve. Errors are reduced. The RedEPTree technique is based on the notion of calculating information gain using entropy and backfitting to minimize variance-induced error [30].

### 3.6. Naive Bayes (NB) Classifier

There are two steps of classified data in the Naive Bayesian approach [31]. The first stage involves evaluating the parameters of a probability distribution using the training input data. In the second stage, the test dataset is categorized based on the greatest posterior probability. The NB classifier's pseudocode is shown below.

### 3.7. AdaBoost

AdaBoost makes it possible to merge various “weak classifiers” into a single classifier which is called “strong classifier.” Decision trees with one level, or decision trees with only one split, are the most popular algorithm used with AdaBoost. Decision Stump is another name for these trees [32]. This approach creates a model by assigning equal weights to all of the data points. It then gives points that are incorrectly categorized with a higher weight. In the next model, all points with greater weights are given more importance. It will continue to train models till a lower error is received [33].

The weight of the training set is used to start the AdaBoost algorithm. Let us consider training set (*x*_1_, *y*),… (*x*_*n*_, *y*_*n*_), in which each *x*_*i*_ is in instance space *X* and each label *y*_*i*_ is in collection of labels Y, that is very much similar to the collection of {−1, +1}. Weight on training instance I on the round *t* is assigned as *D*_It_(*i*). At the start, the same weight is used (*D*_It_(*i*)) = 1/M, *i* = 1,…, M), where It is the iteration number. Then, weight of the misclassified case from the base learning algorithm is then increased in each round. The AdaBoost algorithm's pseudocode is shown below.

And

(4) $$\begin{array}{c} \alpha_{\text{It}}=\frac{1}{2}\text{In}\left[\frac{P_{+1}-P_{-1}}{P_{-1}+P_{-1}}\right]. \end{array}$$

*C*_It_ is the normalization constant, *α*_It_ is used to allow the outcome to be generalized and to solve the problem of overfitting and noise sensitive situations [33]. The real value of *α*_*It*_*h*_*It*_ (*x*) is built using a class probability estimate (P).

## 4. Proposed Methodology

**Figure 1:**
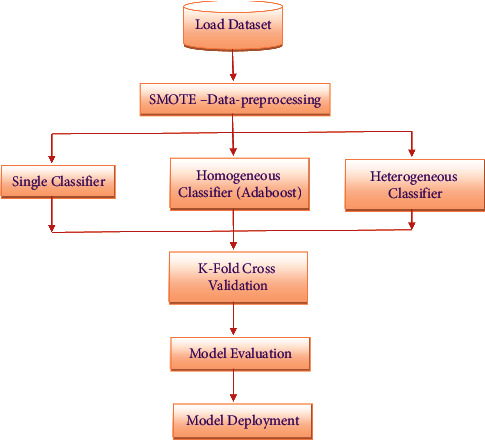
Proposed flow diagram.

The proposed approach contains two phases in this section. SMOTE is used in the initial phase to lessen the impact of data imbalance. Then, the second phase entails classification using Naive Bayes and DT methods (AltDTree, CART, RedEPTree, and RF) [33]. After that, AdaBoost Ensembles of the aforementioned algorithms are constructed and their performance is evaluated. Then, heterogeneous classifiers that are formed by combining two different individual classifiers are evaluated against different performance metrics to figure out the best model.  Figure 1 depicts the flow of the suggested technique.

### 4.1. Dataset

**Figure 2:**
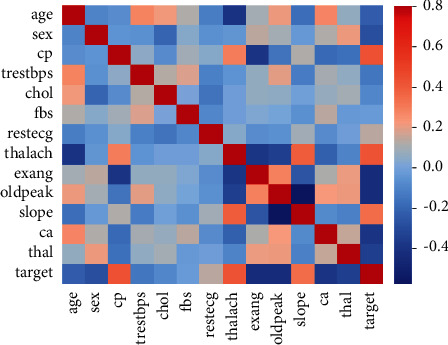
Heatmap depiction of the dataset.

The UCI repository provided the Heart Disease dataset. This dataset comprises 13 medical variables for 304 patients, which helps to determine whether the patient is in the danger of developing heart disease or not, as well as categorize patients who are at risk and those who are not. The pattern that leads to the discovery of patients at risk for heart disease is retrieved from this dataset. There are two aspects to these records: training and testing. Each row corresponds to a single record in this dataset, which has 303 rows and 14 columns.  Table 1 lists all of the qualities and the heatmap is depicted in Figure 2.

### 4.2. Data Preprocessing

Most classification algorithms aim to gather pure samples to learn and make the borderline of each class as definitive as possible in order to perform better prediction. Synthetic instances that are far from the borderline are easier to categorize than those that are near to the borderline, which present a significant learning difficulty for the majority of classifiers. The authors in [32] describe an advanced strategy (A-SMOTE) for preprocessing imbalanced training sets based on these findings. It aims to clearly characterize the borderline and create pure synthetic samples from SMOTE generalization. This approach is divided into two parts, as follows.

**Step 1 .**

The SMOTE technique is used to create a synthetic instance using

(5) $$\begin{array}{c} N=2*\left(r-z\right)+z, \end{array}$$

where *r* denotes majority class samples, *z* denotes minority class samples number, and *N* is the initial synthetic instance number (which is newly generated).

The synthetic instances generated by SMOTE can be approved or rejected based on two criteria, which correspond to the first stage: For example, consider $\hat{x}$ = {${\hat{x}}_{1}$, ${\hat{x}}_{2}$, ${\hat{x}}_{3}$,…. ${\hat{x}}_{N}$} which is the collection of new synthetic instances, and ${\hat{x}}_{i}{}^{\left(j\right)}$ is the *j*th attribute value of ${\hat{x}}_{i}$, *j*∈[1, *M*].Let*S*_*m*_ = {*S*_*m*1_, *S*_*m*2_,… *S*_*mz*_} and *S*_*α*_ = {*S*_*α*1_, *S*_*α*1_, *S*_*α*1_,…*S*_*αr*_} be the set of the minority samples as well as majority samples [32]. In order to make the rejection or acceptance decision, distance is computed between ${\hat{x}}_{i}$ and *S*_*mk*_, *DD*_minority_$\left({\hat{x}}_{i},S_{mk}\right)$ and the distance between ${\hat{x}}_{i}$ and *S*_*αl*_, *DD*_majority_$\left({\hat{x}}_{i},S_{\alpha l}\right)$. For I from N steps, we calculate the distances as stated below, using equations (6) and (7).

(6) $$\begin{array}{c} DD_{\text{minority}}\left({\hat{x}}_{i},S_{mk}\right)=\overset{M}{\underset{j=1}{\sum }}\sqrt{{\left({\hat{x}}_{i}{}^{\left(j\right)}-{\hat{S}}_{mk}{}^{\left(j\right)}\right)}^{2}},\;k\in \left[1,z\right], \end{array}$$

(7) $$\begin{array}{c} DD_{\text{minority}}\left({\hat{x}}_{i},S_{al}\right)=\overset{M}{\underset{j=1}{\sum }}\sqrt{{\left({\hat{x}}_{i}{}^{\left(j\right)}-{\hat{S}}_{al}{}^{\left(j\right)}\right)}^{2}},\;l\in \left[1,r\right]. \end{array}$$

As per (6) and (7), we compute arrays *A*_minority_and *A*_majority_ using (8) and (9).

(8) $$\begin{array}{c} A_{minority}=\left(DD_{\min ority}\left({\hat{x}}_{i},S_{m1}\right),\ldots DD_{\min ority}\left({\hat{x}}_{i},S_{mz}\right)\right), \end{array}$$

(9) $$\begin{array}{c} A_{majority}=\left(DD_{majority}\left({\hat{x}}_{i},S_{a1}\right),\ldots DD_{majority}\left({\hat{x}}_{i},S_{ar}\right)\right). \end{array}$$

Then we choose the minimum value out of *A*_minority_, min(*A*_minority_)and the minimum value out of *A*_majority_, min(*A*_majority_). If *min*(*A*_minority_) is lesser thanmin(*A*_majority_), the new samples are accepted else, rejected.

 
min(*A*_majority_) < min(*A*_majority_) (Accepted).
 
min(*A*_minority_) ≥ min(*A*_majority_) (Rejected).

**Step 2 .**

Then, using the accepted synthetic instances, the following steps are taken to remove the noise.

Suppose $\hat{S}$ = {${\hat{S}}_{1}$, ${\hat{S}}_{2}$, ${\hat{S}}_{3}$,…. ${\hat{S}}_{n}$} is a new synthetic minority received by Step 1. We then compute the distance between ${\hat{S}}_{i}$ with each original minority $S_{m},{\text{Min}}_{\text{Rap}}\left({\hat{S}}_{i},{\hat{S}}_{m}\right)$, defined using

(10) $$\begin{array}{c} S_{m},{\text{Min}}_{\text{Rap}}\left({\hat{S}}_{i}{\hat{S}}_{m}\right)=\overset{z}{\underset{k=1}{\sum }}\overset{M}{\underset{j=1}{\sum }}\sqrt{{\left({\hat{S}}_{i}{}^{\left(j\right)}\;-\;S_{mk}{}^{\left(j\right)}\;\right)}^{2}}, \end{array}$$

where

$S_{m},{\text{Min}}_{\text{Rap}}\left({\hat{S}}_{i}.{\hat{S}}_{m}\right)$ samples rapprochement including all minority and as per (10), *L* is obtained as follows:

(11) $$\begin{array}{c} L=\sum_{i=1}^{n}\left({\text{Min}}_{\text{Rap}}\left({\hat{S}}_{i},S_{m}\right)\right). \end{array}$$

**Step 3 .**

Compute the distance between ${\hat{S}}_{i}$, and each original majority *S*_*a*_, $Maj_{\text{Rap}}\left({\hat{S}}_{i}S_{a}\right)$, described using

(12) $$\begin{array}{c} Maj_{\text{Rap}}\left({\hat{S}}_{i}S_{a}\right)=\overset{r}{\underset{i=1}{\sum }}\overset{M}{\underset{j=1}{\sum }}\sqrt{{\left({\hat{S}}_{i}{}^{\left(j\right)}-S_{al}{}^{\left(j\right)}\right)}^{2}}. \end{array}$$

$Maj_{\text{Rap}}\left({\hat{S}}_{i},S_{a}\right)$ ⟶ samples rapprochement including all majority and as per equation (13) *H* is obtained as follows:

(13) $$\begin{array}{c} H=\sum_{i=1}^{n}\left(Maj_{\text{Rap}}\left({\hat{S}}_{i},S_{a}\right)\right). \end{array}$$

Then, we remove half of synthetic samples which have most likely less distance between ${\hat{S}}_{i}\text{and }S_{a}$ to obtain the data, that is, of high purity.

## 5. Performance Evaluations

The different ML algorithms, namely, Naive Bayes, AltDTree, RedEPTree, CART, and RF, are applied on the dataset as individual classifiers. Their performance is compared in terms of several metrics as described in the next section.

### 5.1. Performance Metrics

If the dataset is not balanced, accuracy may not be a good measure [34]. The number of accurately classified examples divided by total number of data instances is referred to as accuracy. The accuracy is computed using

(14) $$\begin{array}{c} \text{Accuracy}=\frac{TNs+TPs}{TNs+TPs+FPs+FNs}. \end{array}$$

Precision is one of the performance metrics that is going to measure how many correct positive forecasts have been done. So, precision estimates the accuracy of the minority class; then, the ratio of accurately predicted positive instances divided by the total number of positive examples predicted, is used to compute it using

(15) $$\begin{array}{c} \text{Precision}=\frac{TPs}{TPs+FPs}. \end{array}$$

A good classifier should have a precision of 100% (high); only when both numerator and denominator are identical, i.e., TP = TP + FP, can precision become 100% [33].

Recall is a metric that measures how many correct positive predictions were produced out of all possible positive predictions. Unlike precision, which only considers the right positive predictions out of all positive predictions, recall considers the positive predictions that were missed. In this approach, recall provides some indication of the positive class' coverage. The recall is computed using

(16) $$\begin{array}{c} \text{recall}=\frac{TPs}{TPs+FNs}. \end{array}$$

We want both accuracy and recall to be of the value one in a good classifier, which also means FP and FN should be zero. As a result, we require a statistic that considers both precision and recall. The F1-score is a measure that takes precision and recall into account and is defined as follows:

(17) $$\begin{array}{c} F1\text{ Score}=2*\frac{\text{precision}*\text{recall}}{\text{precision}+\text{recall}}. \end{array}$$

To compute error rates in forecasted value, let *P*^*N*^ denote a collection of data having the form (*t*_1_, *r*_1_) , (*t*_2_, *r*_2_),… (*t*_*p*_, *r*_*p*_)such that *t*_*i*_ denotes n-dimensional tuples of test with respective values of *r*_*i*_ for a given response *r* and denotes count of tuples in *P*^*N*^.

In all test instances, the mean-absolute-error (MAE) is the mean of the difference among the projected and guanine value. It is the standard deviation of the prediction error calculated using

(18) $$\begin{array}{c} MAE=\sum_{i=1}^{p}\left|r_{i}-r_{i}{}^{T}\right|. \end{array}$$

The root mean squared error (RMSE) is a well-known approach for calculating numeric prediction success. The mean of the squared discrepancies among every value is computed and its matching true value is used to calculate this value using

(19) $$\begin{array}{c} RMSE=\sqrt{\frac{\sum_{r=1}^{p}{\left(r_{i}-r^{T}{}_{i}\right)}^{2}}{p}}. \end{array}$$

The total absolute mistake is made relative to what the error would have been if the prediction had just been the average of the actual numbers known as Relative Absolute Error (RAE). It is computed using

(20) $$\begin{array}{c} RAE=\frac{\sum_{r=1}^{p}{\left(r_{i}-r^{T}{}_{i}\right)}^{2}}{\sum_{r=1}^{p}{\left(r_{i}-{\bar{r}}_{i}\right)}^{2}}. \end{array}$$

The total squared error made is compared to what the error would have been if the prediction had been the average of the absolute value, known as relative squared error (RRSE). It is computed using

(21) $$\begin{array}{c} RRSE=\sqrt{\frac{\sum_{r=1}^{p}{\left(r_{i}-r^{T}{}_{i}\right)}^{2}}{\sum_{r=1}^{p}{\left(r_{i}-{\bar{r}}_{i}\right)}^{2}}}. \end{array}$$

Table 2 depicts that Random Forest is the best model as it takes only 2.27 seconds for model building (TTBM: Time to Build Model), while the AltDTree has taken 60.18 seconds for model building.

**Figure 3:**
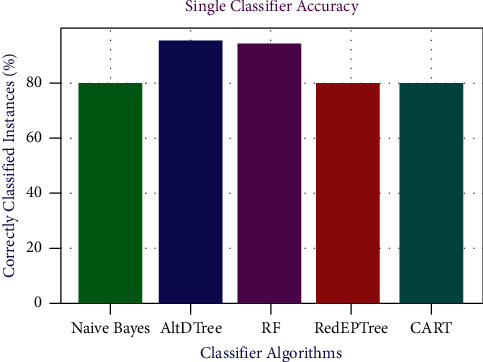
Accuracy prediction for single classifiers.

Figure 3 shows the accuracy forecast for individual classifiers. Among all the aforementioned classifiers being used in the current research work, AltDTree provides the best accuracy of 93.56%. Random Forest provides 92.45% accuracy and NB classifier prediction is the lowest with 78.67% accuracy.

**Figure 4:**
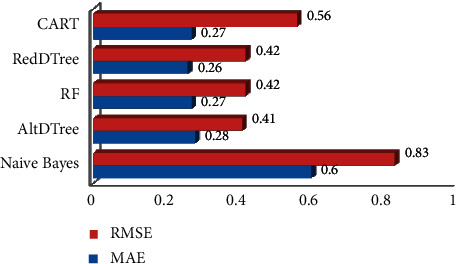
Error rates of individual classifier.

Figure 4 depicts the rates of errors obtained from the individual classifiers. AltDTree MAE rate is 0.28 and RMSE rate value is 0.41. This demonstrates that there is low error recorded during the prediction procedures. However, NB has a higher error rate, i.e., 0.60 MAE and 0.83 RMSE, respectively.

Table 3 demonstrates that AdaBoost-RF is the best model, as it has taken only 10.34 seconds to build the model. But the AdaBoost-CART is the worst model as it takes 295.45 seconds to build the model. Also, AdaBoost-RF has highest F1-value of 0.98 and AdaBoost-NB has the lowest F1-value of 0.81.

**Figure 5:**
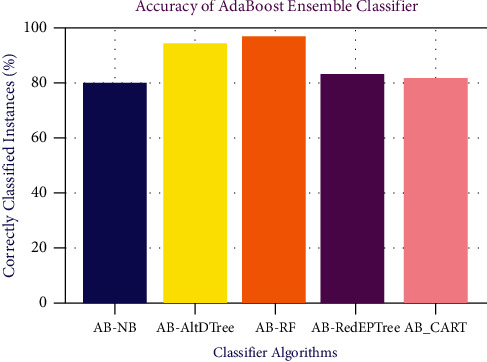
Accuracy of AdaBoost classifier.

From Figure 5, AdaBoost-RF predictions are better than any other mentioned classification algorithm with an accuracy of 95.47%. However, AdaBoost-AltDTree provides 93.56% prediction accuracy and stands second. The AdaBoost-NB provides the least prediction rate of 80.6%.

**Figure 6:**
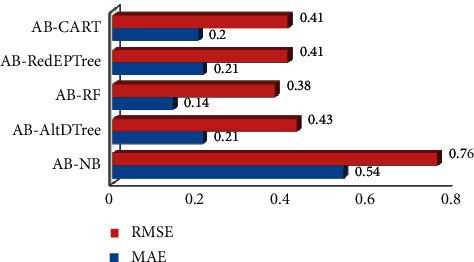
AdaBoost classifier error rate.

Figure 6 depicts the different error rates that were recorded. AdaBoost Ensemble classifiers provide the lowest error rate of 0.14 for MAE and 0.38 for RMSE. However, AdaBoost-NB has a higher error rate, i.e., 0.54 and 0.76 for MAE and RMSE, respectively, whose values are almost the same as that of NB individual classifier.

Table 4 depicts the results of ensemble classifiers which are heterogeneous in nature. RF-CART and RF-RedEPTree take 7.34 seconds and 7.89 seconds for building the model, respectively, which are very low. However, AltDTree-CART has taken 598.02 seconds being the worst time for building the model. So, it can be said that RF-CART has a higher F1-score of 0.85, and RF-RedEPTree is second with F1-score of 0.84. AltDTree-RF and AltDTree-CART have the worst F1-scores of 0.68 and 0.69, respectively.

**Figure 7:**
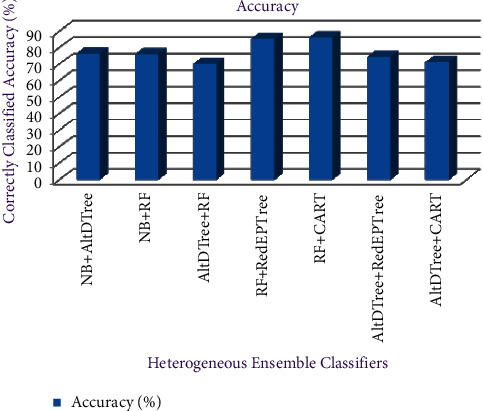
Accuracy of heterogeneous ensemble classifiers.

From Figure 7, RF-CART provides the best accuracy of 86.29% in comparison to others, followed by RF-RedEPTree with 85.45% prediction accuracy. AltDTree-RF has the lowest accuracy value of 70.12%.

**Figure 8:**
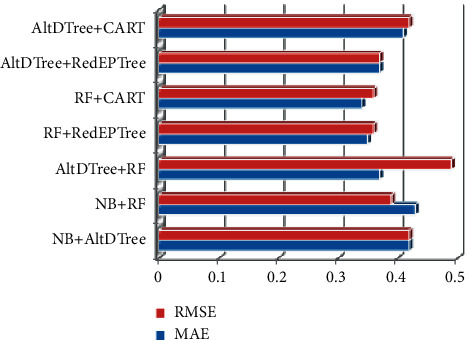
Error rates for heterogeneous ensemble classifiers.

Figure 8 depicts error rates obtained by ensemble classifiers are heterogeneous in nature. RF-CART exhibits the lowest error rate of 0.34 (MAE) and RMSE of 0.36. However, NB-RF has the highest MAE rate of 0.43 and AltDTree-RF has the highest RMSE rate of 0.49.

## 6. Conclusion

The AdaBoost Ensemble model for heart disease prediction has been proposed in this work, which is based on recognized feature patterns. In the diagnosis of cardiac disease, it can be compared with classic data mining methods. Ensemble classification approaches replace traditional methods of extracting meaningful information during the feature extraction step. The homogeneous classifiers and ensemble classifiers which are formed by combining multiple methods called heterogeneous classifiers were employed in this study. The data mining preprocessing technique using Synthetic Minority Oversampling Technique (SMOTE) is used to cope with the problem of class imbalance as well as noise present in the heart disease dataset. The best time to build the model for heterogeneous ensemble classifiers is 7.34 seconds for RF-CART and 7.89 seconds for RF-RedEPTree ensemble, according to the experimental results. NB-AltDTree has been observed to have taken the worst time of 598.02 seconds to build the model. With 86.29% prediction accuracy, RF-CART outperforms other classification algorithms, followed by RF-RedEPTree with 85.45% prediction accuracy. As per the results, AdaBoost-RF classifier exhibits 0.14 error rate for MAE which is the lowest and 0.38 for RMSE among the other AdaBoost Ensemble classifiers. In all the overall experiments, the performances of classifiers were compared, and the findings revealed that AdaBoost-RF is the best among other classifiers with 95.47% accuracy.

## Figures and Tables

**Algorithm 1:**
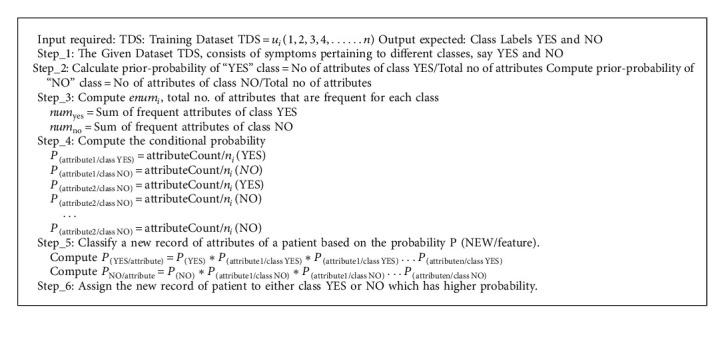
Pseudocode of NB classifier.

**Algorithm 2:**
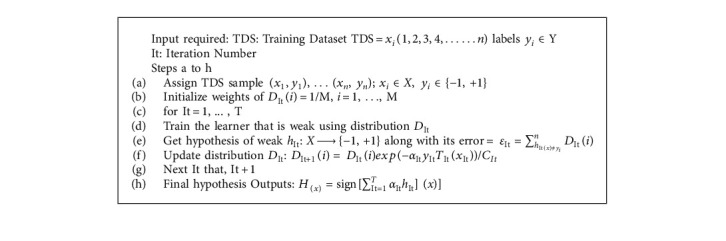
Pseudocode of AdaBoost classifier.

**Table 1:** Attributes of the dataset.

Sl. No.	Features	Description	Values
1	Age	Age in years	Continuous
2	Sex	Gender of patient	Male/female
3	CP	Chest pain	Four types
4	Trestbps	Resting blood pressure	Continuous
5	Chol	Serum cholesterol	Continuous
6	FBS	Fasting blood sugar	<, or >120 mg/dl
7	Restecg	Resting electrocardiograph	Five values
8	Thalach	Maximum heart rate achieved	Continuous
9	Exang	Exercise induced angina	Yes/no
10	Oldpeak	ST depression when working out compared to the amount of rest taken	Continuous
11	Slope	Slope of peak exercise ST segment	Up/flat/down
12	Ca	Gives number of major vessels colored by fluoroscopy	0–3
13	Thal	Defect type	Reversible/fixed/normal
14	Num (disorder)	Heart disease	Not present (“NO”)/present in the four major types (“YES”)

**Table 2:** Single classifier evaluation comparison.

Performance metrics	Naive Bayes	AltDTree	RF	RedEPTree	CART
TTBM (sec)	4.56	60.18	2.11	10.25	52.24
Accuracy (%)	78.6	93.56	92.45	79.23	78.67
MAE	0.60	0.28	0.27	026	0.27
RMSE	0.83	0.41	0.42	0.42	0.56
RAE	120	67.71	77.87	79.12	68.91
RRSE	127.41	95.33	82.92	97.89	98.34
F1-score	0.3	0.85	0.84	0.83	0.81

**Table 3:** AdaBoost classifier.

Performance metrics	AB-NB	AB-AltDTree	AB-RF	AB-RedEPTree	AB-CART
TTBM (sec)	18.32	30.01	10.34	64.35	295.45
Accuracy (%)	80.6	93.56	95.47	82.23	81.67
MAE	0.54	0.21	0.14	0.21	0.20
RMSE	0.76	0.43	0.38	0.41	0.41
RAE	129.79	57.78	35.87	45.19	41.61
RRSE	155.62	96.23	65.47	91.03	91.08
F1-score	0.81	0.94	0.98	0.83	0.87

**Table 4:** Ensemble classifiers, heterogeneous.

Performance metrics	NB + AltDTree	NB + RF	AltDTree + RF	RF + RedEPTree	RF + CART	AltDTree + RedEPTree	AltDTree + CART
TTBM (sec)	30.03	32.05	398.12	7.89	7.34	357.77	598.02
Accuracy (%)	76.45	76.05	70.12	85.45	86.29	74.49	71.29
MAE	0.42	0.43	0.37	0.35	0.34	0.37	0.41
RMSE	0.42	0.39	0.49	0.36	0.36	0.37	0.42
RAE	99.23	92.23	80.12	71.01	70.89	73.23	89.23
RRSE	98.23	97.49	101.22	91.29	90.12	93.37	99.34
F1-score	0.74	0.75	0.68	0.84	0.85	0.73	0.69

## Data Availability

The [UCI repository] data used to support the findings of this study are included within the article.
